# Effectiveness of polyethylene glycol and glutaraldehyde as enhancers for lipase-immobilized hybrid organic–inorganic nanoflowers

**DOI:** 10.1007/s00449-025-03181-x

**Published:** 2025-06-05

**Authors:** Jessica Siew Kiong Ling, Sie Yon Lau, Shamini Anboo, Murat Yılmaz

**Affiliations:** 1 Department of Chemical Engineering, Faculty of Engineering and Science, Curtin University, 98009 Miri, Sarawak Malaysia; 2 Curtin Malaysia Research Institute, Curtin University, 98009 Miri, Sarawak Malaysia; 3 Department of Chemistry and Chemical Processing Technologies, Bahçe Vocational School, Osmaniye Korkut Ata University, 80000 Osmaniye, Turkey

**Keywords:** Lipase hybrid nanoflowers, Polyethylene glycol, Glutaraldehyde

## Abstract

The present study investigates the influence of polyethylene glycol (PEG) and glutaraldehyde (GA) on the synthesis and enzymatic activity of lipase hybrid nanoflowers. The effect of lipase concentration on hybrid nanoflower formation was first assessed, revealing that the optimum lipase concentration was 0.2 mg/mL. At this concentration, the encapsulation of lipase within the hybrid nanoflowers reached its maximum efficiency. Further, the effects of PEG and GA concentrations, as well as pH, on the enzymatic activity of the nanoflowers were evaluated. The results demonstrated that 2% (v/v) PEG and 3% (v/v) GA were the most effective concentrations, with the highest activity observed at pH 8. Comparative studies showed that GA-treated lipase hybrid nanoflowers exhibited a remarkable 160% increase in enzymatic activity over the free lipase, outperforming PEG in terms of catalytic performance. Scanning Electron Microscopy (SEM) and Fourier Transform Infrared (FTIR) spectroscopy analyses confirmed that both PEG and GA treatments altered the morphology and structural characteristics of the hybrid nanoflowers, with GA inducing more pronounced changes. Despite these morphological alterations, the enzymatic activity was significantly enhanced, particularly in the GA-treated hybrid nanoflowers. In conclusion, this study highlights the superior performance of glutaraldehyde as an enhancer for the production of highly active lipase hybrid nanoflowers, offering promising applications in biocatalysis and enzyme immobilization.

## Introduction

Enzymes have been widely implemented in various industries, such as pharmaceuticals, food, agriculture, materials and polymers, fragrances and flavors, and fine chemicals [1–3]. They act as biocatalysts to speed up the rate of chemical reactions by providing an alternative path that requires lower activation energy, thereby enabling bulk production at lower operating costs. However, due to their high cost, the recovery and reusability of enzymes are highly desirable for continuous industrial processes. The recovery and reusability of conventional free enzymes, however, are limited due to their hydrophilic properties, which complicate the enzyme separation process, as well as their low resistance to environmental changes, which makes them unstable [4]. To mitigate such problems, immobilization techniques to fix the enzymes within solid supports or carriers are introduced, via physical methods such as adsorption and entrapment, and chemical methods such as cross-linking and covalent binding [4–7]. These techniques lead to more robust and cost-effective heterogeneous enzyme systems that are highly recyclable. However, challenges arise when immobilized enzymes exhibit a significant reduction in enzymatic activity compared to free enzymes. This is due to changes in enzyme conformation, which block some of the active sites, making them inaccessible for substrate binding [8]. Consequently, the loss of enzymatic activity results in a lower reaction rate. Therefore, a simple solution to increase enzyme stability while retaining enzymatic activity is highly desirable.

**Fig. 1:**
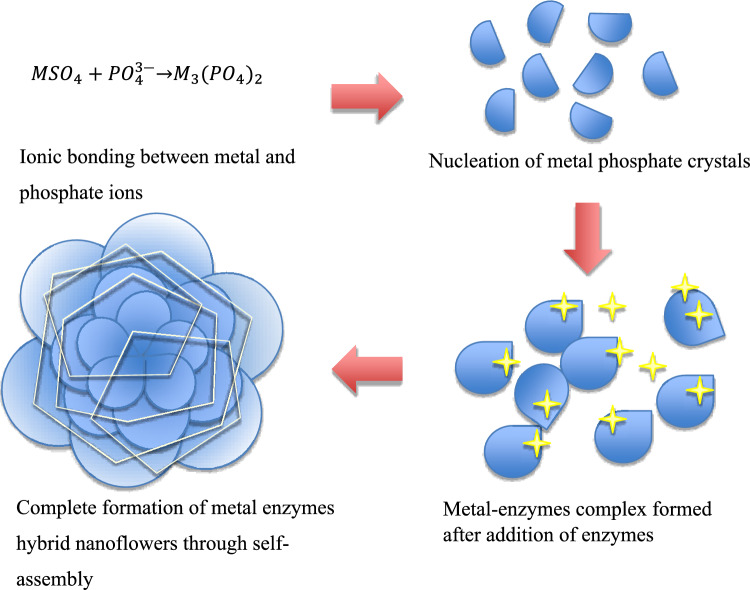
Mechanism of hybrid nanoflowers formation

One of the recent advanced immobilization methods using nanomaterials as solid supports has been proposed to compensate for the loss of enzymatic activity [8]. In addition to common nanoscale features, the specific surface area is a key characteristic that defines nanomaterials, typically ≥ 60 m^2^/cm^3^ [9]. Nanomaterials have been extensively applied in biocatalysis, biosensors, bioanalytical devices, and drug delivery since 2000, particularly nanoflowers, due to their hierarchical flower-like structure, which provides a high surface area to volume ratio, thus promoting a higher reaction rate compared to conventional nanoparticles [10, 11]. Alongside other distinctive nanostructures, aesthetically pleasing flower-like nanomaterials (NFs) have garnered significant attention in recent years due to their exceptionally high specific surface area. This characteristic enhances the efficiency of surface interactions, including strong adsorption and high loading capacity, making NFs suitable candidates for enzyme immobilization [12]. Recently, researchers have employed hierarchical nanoflowers (NFs) for enzyme immobilization. In most cases, compared to free enzymes, these NF-based immobilized enzymes demonstrate enhanced catalytic activity and stability [13, 14]. The combination of enzymes and metal cations through the nanoscale process results in the formation of organic–inorganic hybrid nanoflowers, where ‘organic’ refers to the enzymes and ‘inorganic’ to the metal ions. In 2012, the first protein-inorganic hybrid nanoflower was accidentally discovered by Ge, Lei, and Zare [15]. Figure 1 illustrates the underlying mechanism of organic–inorganic hybrid nanoflower formation. Initially, the metal cations form strong ionic bonds with the phosphate anions, leading to the nucleation of primary metal phosphate crystals. Next, the nitrogen atoms in the amide groups of the enzymes form complexes with the metal ions. Starting at the enzyme backbone, the petals grow from the bonded metal ions, continuing until the complete formation of the hybrid nanoflowers [15]. The layered petals, with their large surface area, allow the substrate to bind more efficiently to the embedded enzymes, thus compensating for the activity loss due to immobilization. However, the activity is only maintained for a single reaction cycle. When the hybrid nanoflowers are subjected to multiple recycling processes, enzymatic activity is found to drop drastically [16]. This is due to the enzyme backbone being damaged during repeated use and washing. Once the metal ions that form the petals lose their backbone, the morphology is also disrupted. To address this, researchers recommend using a cross-linking agent to further strengthen the intermolecular forces between the enzymes and metal ions [16]. As a result, enzyme hybrid nanoflowers with superior properties—such as high stability, high enzymatic activity, and high reusability—have been successfully developed.

Lipases, classified as triacylglycerol acyl hydrolases (EC: 3.1.1.3), are ubiquitous enzymes produced by various organisms, including plants, insects, animals, and microbes, with fungi, yeasts, and bacteria being the primary sources [17]. Yeasts and fungi serve as primary and cost-effective sources for lipase production [18]. This enzyme belongs to the hydrolase class, which is known for catalyzing the hydrolysis of ester bonds, including those found in certain widely used polymers. Abdel-Mageed and co-workers investigated the possibility of producing a stable biocatalyst with lipase enzyme nano-powder using the nano spray dryer BÜCHI B-90 [19]. Additionally, they obtained a sustainable and robust biocatalyst for ultrasound-assisted olive oil hydrolysis via immobilization of *Mucor racemosus* lipase [20].

Plastic waste pollution has become a major environmental challenge due to the rapid growth in plasticware demand, particularly during the COVID-19 pandemic, which has also led to an increase in plasticware disposal. According to the latest Malaysian environmental statistics, around 40 thousand metric tonnes of clinical waste were generated in 2020, primarily consisting of self-test kits and personal protective equipment, such as face masks, gloves, face shields, and protective gowns, due to the outbreak of COVID-19 [21]. These items are all made of plastics and are not ideal for reuse or recycling due to public health considerations. The complete degradation of plastics in the environment can take more than 1000 years [22]. Hence, the development of lipase-copper hybrid nanoflowers is considered a promising solution for plastic degradation applications. While lipase hybrid nanoflowers can accelerate plastic degradation, further enhancement of lipase activity is desired to boost the degradation process and shorten the time required for complete degradation. Lipase enzymes play a critical role in the hydrolysis of ester bonds, which are the primary linkages in synthetic polymers such as polyethylene terephthalate (PET). By cleaving these ester bonds, lipases facilitate the depolymerization of polymers into smaller, more biodegradable molecules, offering an eco-friendly solution for plastic waste management [23, 24]. However, the application of free lipase is often limited by inadequate stability, poor reusability, and sensitivity to adverse environmental conditions. Immobilizing lipase, particularly within hybrid nanoflowers, offers several advantages, including enhanced stability, increased catalytic activity, and improved resistance to denaturation—factors essential for prolonged and industrial-scale plastic degradation. Additionally, immobilized lipase enables efficient recovery and reuse, significantly reducing operational costs and increasing the sustainability of the process. The hybrid nanoflower matrix also imparts structural rigidity, protecting the enzyme from mechanical stress during plastic breakdown. Therefore, exploring immobilized lipase systems represents a promising approach for the efficient and environmentally sustainable degradation of persistent plastic pollutants such as PET [23, 24]. In their study on the biodegradation of synthetic plastics by *Aspergillus niger* extracellular lipase, Safdar et al. observed that the enzyme they obtained degraded PE, PET, and PS samples within a 30-day incubation period [23]. Khan et al. studied the enzyme-embedded degradation of Poly(*ε-caprolactone*) using lipase derived from Probiotic *Lactobacillus plantarum* and found that the mass losses observed with 2%, 4%, 6%, and 8% lipase-embedded PCL films were 11%, 27%, 32% and 35% after 2 days, respectively [24]. Immobilization of lipase on nanoflowers often results in a slight decrease in lipase activity [15]. To further enhance the performance of lipase hybrid nanoflowers, cross-linking agents such as PEG and glutaraldehyde are commonly employed during the synthesis process.

Polyethylene glycol (PEG) is a water-soluble polymer that exhibits compatibility with a wide range of organic solvents. It consists of repeated ethylene glycol monomer units, and its properties are influenced by the molecular weight. PEG can exist in two forms: hydrophilic or amphipathic, with the latter possessing both hydrophilic and hydrophobic characteristics. The hydrophilic nature of PEG is attributed to the presence of hydroxyl groups (-OH), which enable the formation of hydrogen bonds, while the hydrophobicity is governed by the long alkyl chains (–CH_2_–CH_2_) [25]. The molecular weight of PEG plays a critical role in determining its properties. At lower molecular weights, such as PEG-600, the polymer tends to be hydrophilic because the short alkyl chains allow the hydroxyl groups to dominate. Conversely, at higher molecular weights, such as PEG-20,000, the hydrophobic effect of the long alkyl chains becomes more prominent, rendering the polymer amphipathic [25]. Consequently, PEG at higher molecular weights exhibits both hydrophilic and hydrophobic properties. The process of conjugating PEG to proteins, known as PEGylation, can occur through either covalent or non-covalent interactions, leading to protein precipitation [26]. PEG of low molecular weight typically forms hydrogen bonds with proteins, whereas higher molecular weight PEG interacts strongly with the hydrophobic surfaces of proteins [25–27]. To form stable covalent bonds with protein amide nitrogen atoms, PEG often requires activation using aromatic compounds such as cyanuric chloride, p-nitrophenyl chloroformate, succinimidyl succinate, and succinimidyl carbonate [28–30].

Glutaraldehyde is a type of homobifunctional reagent that exists in various forms (monomers, oligomers, and polymers) in equilibrium. It typically consists of dialdehyde functional groups that are highly reactive toward the amino groups of proteins (such as lipase), promoting the formation of a chemically modified, stable structure via covalent cross-linkages (Schiff base reaction). For polymeric structure of glutaraldehyde, cross-linking can also occur between unsaturated C=C bonds and the amino group of proteins through Michael-type addition reaction [31]. Compared to physical modification, the covalent bonding induced by glutaraldehyde is much stronger and is, in fact, the strongest type of bond among intramolecular forces. Hence, glutaraldehyde is frequently used as a cross-linker to enhance the stability and rigidity of the enzyme matrix.

The incorporation of PEG and GA with lipase immobilization on nanoflowers is believed to further enhance the enzymatic activity of lipase hybrid nanoflowers through a combined effect from both agents. However, due to the distinct properties of both PEG and GA, it is difficult to predict which one will impart better performance. Therefore, a comparative study between PEG and GA is needed to gain a better understanding of their effects on lipase hybrid nanoflowers. To date, no literature has reported a comparison between PEG and glutaraldehyde as enhancers for enzyme hybrid nanoflowers. This study presents a novel approach by systematically evaluating the synergistic effects of polyethylene glycol (PEG) and glutaraldehyde (GA) pretreatment on the activity and morphology of lipase-copper hybrid nanoflowers. The originality lies in optimizing both hydrophilic (PEG) and covalent cross-linking (GA) modifiers to enhance enzymatic performance, providing valuable insights into the structural–functional relationship of hybrid nanomaterials for biocatalytic applications. Therefore, in this work, the performance of PEG and glutaraldehyde will be investigated and compared in lipase-copper hybrid nanoflowers, along with determining the optimum parameters, including the concentrations of lipase, PEG, GA, and pH.

## Experimental section

### Materials

The type of lipase used is porcine pancreas lipase. Sodium phosphate dibasic, copper (II) sulfate anhydrous, and n-heptane are procured from Merck (Darmstadt, Germany). Bovine serum albumin, Bradford reagent, glutaraldehyde, cupric acetate monohydrate, pyridine, and triton X-100 are purchased from Sigma-Aldrich (M) (Selangor, Malaysia). Sodium phosphate monobasic and polyethylene glycol 600 are obtained from Acros Organics (Geel, Belgium). The olive oil is purchased from the local market in Miri (Sarawak, Malaysia).

### Synthesis of lipase hybrid nanoflowers

A 2 mL of 120 mM aqueous copper (II) sulfate (CuSO_4_) is added to 120 mL of 0.1 M sodium phosphate buffer (pH 7.6) with 0.05 mg/mL lipase. The mixture is sonicated at 40 kHz for 7 min, followed by centrifugation at 4000 rpm for 5 min at 4 $^\circ{\rm C}$ with a refrigerated centrifuge (Universal 320R, Hettich). The blue precipitates are collected and freeze-dried for 24 h using a freeze dryer (Fisher 1.5 L, Fisher Labconco). The lyophilized lipase-Cu hybrid nanoflowers are stored in a freezer at 4 $^\circ{\rm C}$. The steps are repeated for different concentrations of lipase with 0.1, 0.2, 0.5, and 1.0 mg/mL [16, 32].

### Synthesis of treated-lipase hybrid nanoflowers

An optimum concentration of lipase (0.2 mg/mL) is added to 0.1% (v/v) glutaraldehyde or PEG in 0.1 M sodium phosphate buffer (pH 7.6). The mixture is shaken for 60 min at room temperature using an orbital shaker (KS501, IKA). After that, the treated lipase is readily used for the synthesis of hybrid nanoflowers. A 2 mL of 120 mM aqueous copper (II) sulfate (CuSO_4_) is directly added to the treated lipase solution. The rest of the procedure follows the same as stated previously for free lipase. The steps are repeated at 0.5, 1.0, 2.0, and 3.0% (v/v) for both cross-linkers. For the pH study, the pH is varied from pH 4–9 with concentrations of PEG and glutaraldehyde fixed at optimum [32–34].

### Encapsulation yield of lipase

The concentration of the lipase left in the supernatant is measured via the Bradford assay method. Bovine serum albumin (BSA) dilutions with concentrations between 0.01 and 0.5 mg in 1 mL of 0.1 M sodium phosphate buffer (pH 7.6) are prepared as standards to obtain a calibration curve. Sodium phosphate buffer is used as a blank. The 10% (v/v) BSA standards and samples are mixed with Bradford reagent in cuvettes, followed by measurement of absorbance at 595 nm after 5 min of mixing via UV–Vis spectrophotometer (Type Lambda 25 UV/VIS Double Beam, Perkin Elmer). The percentage difference of total lipase concentration and lipase left in the supernatant gives encapsulation yield of lipase inside the hybrid nanoflowers [35, 36]. The encapsulation yield of lipase can be calculated by using Eqs. 1–2.

1 $$Untrapped lipase \left[\frac{mg}{mL}\right]=\frac{Average absorbance}{Slope of calibration curve}$$

2 $$Percentage immobilization yield \left[\%\right]=\frac{Total lipase -Untrapped lipase}{Total lipase }\times 100$$

### Enzymatic activity analysis

By referring to Mustafa et al., the lipase assay is performed via colorimetric method using olive oil as the substrate, with some modifications [37]. The assay reagent, cupric acetate pyridine reagent (CAPR) is prepared by dissolving 8.244 g of cupric acetate monohydrate (7.5% (w/v) cupric acetate) in distilled water. Pyridine is added until the reagent reaches pH 6. The substrate emulsion is prepared by mixing an equal volume of olive oil, triton X-100, and 0.1 M sodium phosphate buffer, with a diluted concentration of triton X-100 (10% v/v) in sodium phosphate buffer. To construct the calibration curve, oleic acid standards are prepared with concentrations ranging from 1 to 10 mM in 1.9 mL of n-heptane. For lipase assay, 20 mg of the samples are dissolved in 5 mL sodium phosphate buffer and allowed to react with an equal volume of olive oil emulsion for 10 min. A 0.5 mL of the reaction mixture is extracted and added to a test tube with 1.9 mL of n-heptane and 0.6 mL of CAPR. The test tube is shaken vigorously, and the upper layer is transferred into cuvettes. Absorbance is read at 655 nm using a UV–Vis spectrophotometer (Type Lambda 25 UV/VIS Double Beam, Perkin Elmer) with n-heptane as the blank. The amount of n-heptane used should be increased for dilution if the absorbance reading is high.

The total lipase activity is defined as unit (U), where one unit (µmol/min) equals the amount of oleic acid (µmol) produced over reaction time (mins). The specific lipase activity (U/g) is defined as the total lipase activity that can be achieved by one gram of lipase enzyme.

### Characterization of lipase hybrid nanoflowers

The synthesized lipase-copper hybrid nanoflowers with and without treatment are analyzed with a scanning electron microscope (SEM) to examine the morphology and Fourier transform infrared (FTIR) spectrophotometer (Cary 630, Agilent) to confirm the stretching and vibrations of certain functional groups.

### Bioplastic degradation

To prepare starch based bioplastic, the mixture of 1 g corn starch, 7 mL water, and 0.7 mL of each 5% (v/v) acetic acid and glycerol is cooked on a hotplate until thicken. The mixture is poured on agar plate and swirled evenly, then dried overnight in oven at 50 $^\circ{\rm C}$. The bioplastic is cut into small pieces weighing about 0.1 g. For degradation test, the bioplastic pieces are added to conical flasks containing 50 mL phosphate buffer of pH 8 and 20 mg of untreated, 2% PEG treated, and 3% GA treated lipase hybrid nanoflowers respectively. The test is carried out in a digital shaking incubator of 40 $^\circ{\rm C}$ for 7 days at 200 rpm. Weight is recorded before and after degradation to compute for percentage degradation.

### Statistical analysis

All experiments are repeated at least twice and results are computed as average values.

## Results and discussion

### Synthesis of lipase hybrid nanoflowers

The immobilization of lipase within nanoflowers is done by mixing 120 mM copper (II) sulfate solution into the sodium phosphate buffer containing lipase enzyme. The lipase concentration is varied at 0.05, 0.1, 0.2, 0.5, and 1.0 mg/mL. To study the optimum lipase concentration that renders the highest encapsulation yield within hybrid nanoflowers, the unembedded lipase left in the supernatant after centrifugation is analyzed through the Bradford assay. Before this, a standard calibration curve is needed for protein quantification analysis, where BSA is chosen as the standard. The percentage encapsulation yield of lipase inside nanoflowers is computed in Table 1.

**Table 1:** Percentage yield of lipase embedded in nanoflowers

Total lipase concentration [mg/mL]	Absorbance at 595 nm				Amount of untrapped lipase [mg/mL]	Percentage yield of lipase immobilized [%]
	1	2	3	Average		
0.1	0.0225	0.0219	0.0208	0.0217	0.0197	80.3
0.2	0.0105	0.0132	0.0121	0.0119	0.0108	94.6
0.5	0.0627	0.0643	0.0627	0.0632	0.0573	88.5
1.0	0.1407	0.1389	0.1404	0.14	0.1268	87.3

Based on the results, the increasing trend of percentage encapsulation yield of lipase can be observed until reaching the maximum saturation point of lipase concentration at 0.2 mg/mL. Beyond the saturation limit at the constraint of 120 mM copper (II) ions, no more lipase can be immobilized into the nanoflowers, and hence, the amount of untrapped lipase left in the supernatant increases. Consequently, the percentage immobilization yield decreases. In this study, the optimum lipase concentration is 0.2 mg/mL at 120 mM copper (II) ions. The highest percentage of lipase encapsulation achieved is 94.6%. The result is comparable with Anboo et al.’s current work which showed 0.3 mg/mL as the optimum lipase concentration at 200 mM copper (II) sulfate [38].

### Synthesis of treated-lipase hybrid nanoflowers

**Fig. 2:**
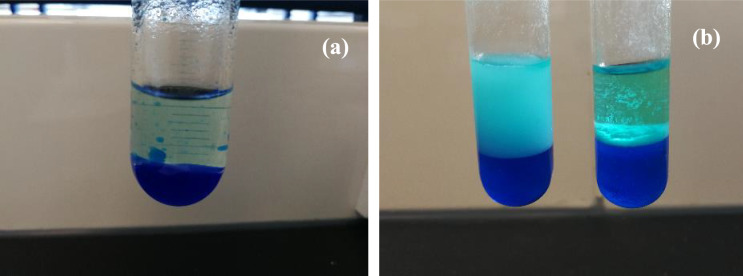
Assay mixture for oleic acid (**a**) and hybrid nanoflowers samples (**b**)

To examine the optimum concentration of additives, similar parameters are studied at 0.1, 0.5, 1.0, 2.0, and 3.0% (v/v) for both PEG and GA to allow direct comparison of their effect on the lipase catalytic performance. By using the optimum lipase concentration of 0.2 mg/mL as determined from the previous section, the free lipase is treated with different concentrations of PEG or GA for 60 min, followed by the addition of 120 mM copper (II) sulfate directly into the buffer solution containing the treated lipase to produce PEG or GA treated lipase-copper hybrid nanoflowers. The lipase activity of different conditions is assayed via colorimetric copper-soap method, which is expressed in terms of micromole of oleic acid produced within one minute (µmol/min), in short, known as one unit (U). Before the lipase assay, it should be ensured that the olive oil emulsion is fully homogenized by stirring with a magnetic bar. Otherwise, the emulsion can be separated into two layers easily in a short while, which may result in large errors. 20 mg of each treated sample is dissolved in phosphate buffer solution (pH 7.6) and reacts with the well-dispersed olive oil emulsion. After a certain reaction time, the enzyme–substrate mixture is shaken vigorously with CAPR and n-heptane, so that the product, oleic acid reacts with CAPR to form copper soaps, which is later extracted by the solvent into the upper layer (Fig. 2). As compared to oleic acid, the hybrid nanoflowers seem to impart opaque blue colour instead of transparent green colour. Surprisingly, after nearly one month, the appearance of a clear green colour layer is observed which might be due to deterioration in the emulsification effect of triton X-100, which decreases the turbidity.

**Fig. 3:**
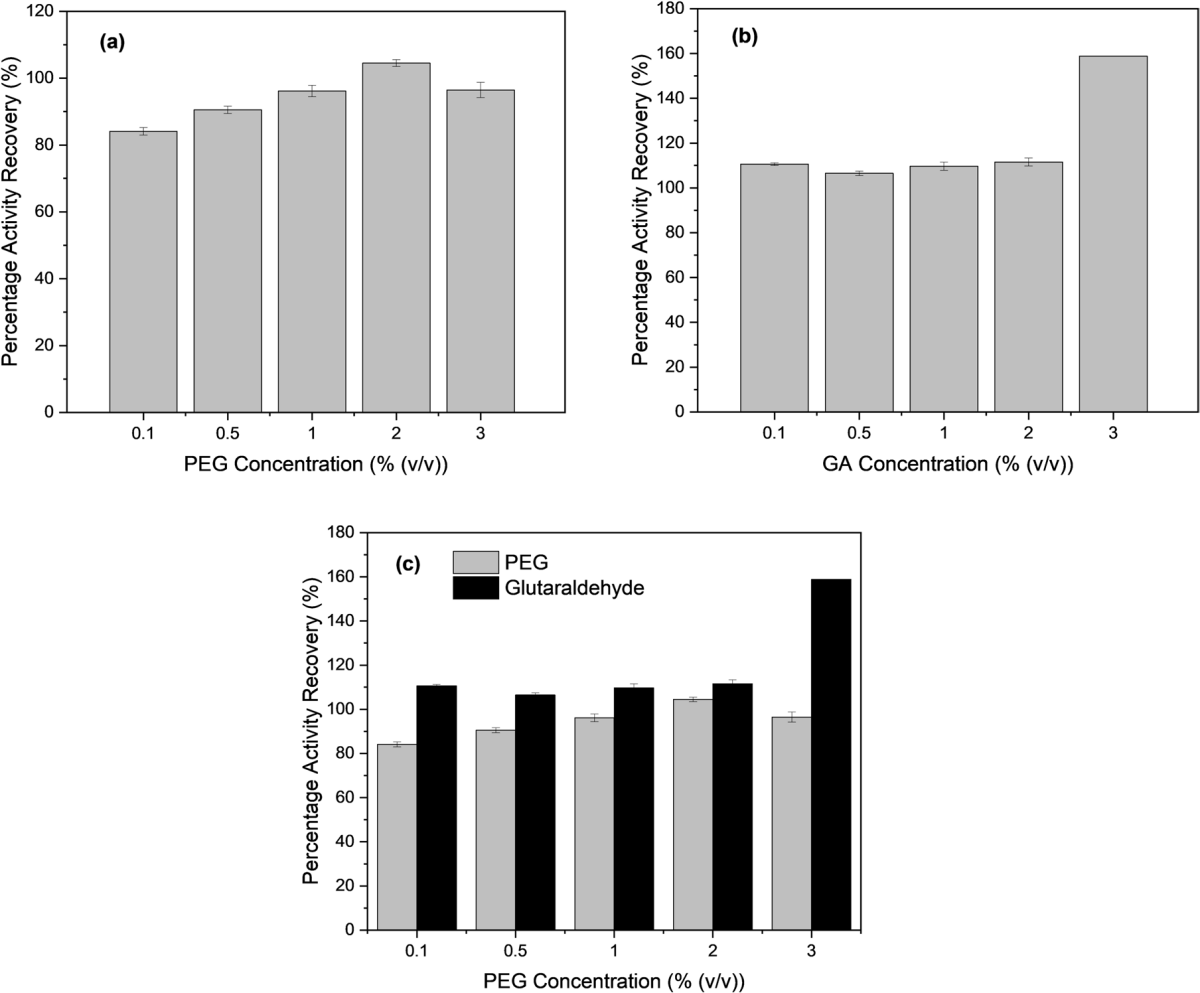
Percentage activity recovery at different PEG concentrations (**a**), GA concentrations (**b**), and percentage activity recovery of PEG vs GA (**c**)

The optimum PEG concentration is found to be 2% (v/v) which exhibits the highest activity performance, as presented in Fig. 3a. Further, an increase in PEG concentration will lead to a reduction in lipase activity, which is similar to the trend proposed by Souza et al. [39]. Meanwhile, by observing Fig. 3b, the lipase activity of GA-treated lipase hybrid nanoflowers shows a boost up when GA concentration is increased to 3% (v/v). Figure 3c presents the comparison of the catalytic activity of PEG and GA at different concentrations. From the comparison, it is found that GA-treated lipase hybrid nanoflowers perform better catalytic activity than PEG for all the concentration parameters investigated. Hence, it can be concluded that glutaraldehyde is a better enhancer than PEG in terms of lipase activity performance, particularly at 3% (v/v) which imparts approximately 160% improvement.

### Effect of pH

The effect of pH is also examined on both PEG and GA-treated lipase hybrid nanoflowers by fixing the lipase concentration at 0.2 mg/mL, PEG concentration at 2% (v/v), and GA concentration at 3% (v/v). Sodium phosphate buffer is prepared at pH 4, 6, 7, 8, and 9 from sodium phosphate monobasic dihydrate and sodium phosphate dibasic. Similarly, for the catalytic activity study, the olive oil emulsion is required to be prepared at this range of pH since sodium phosphate buffer is used as one of the ingredients.

**Fig. 4:**
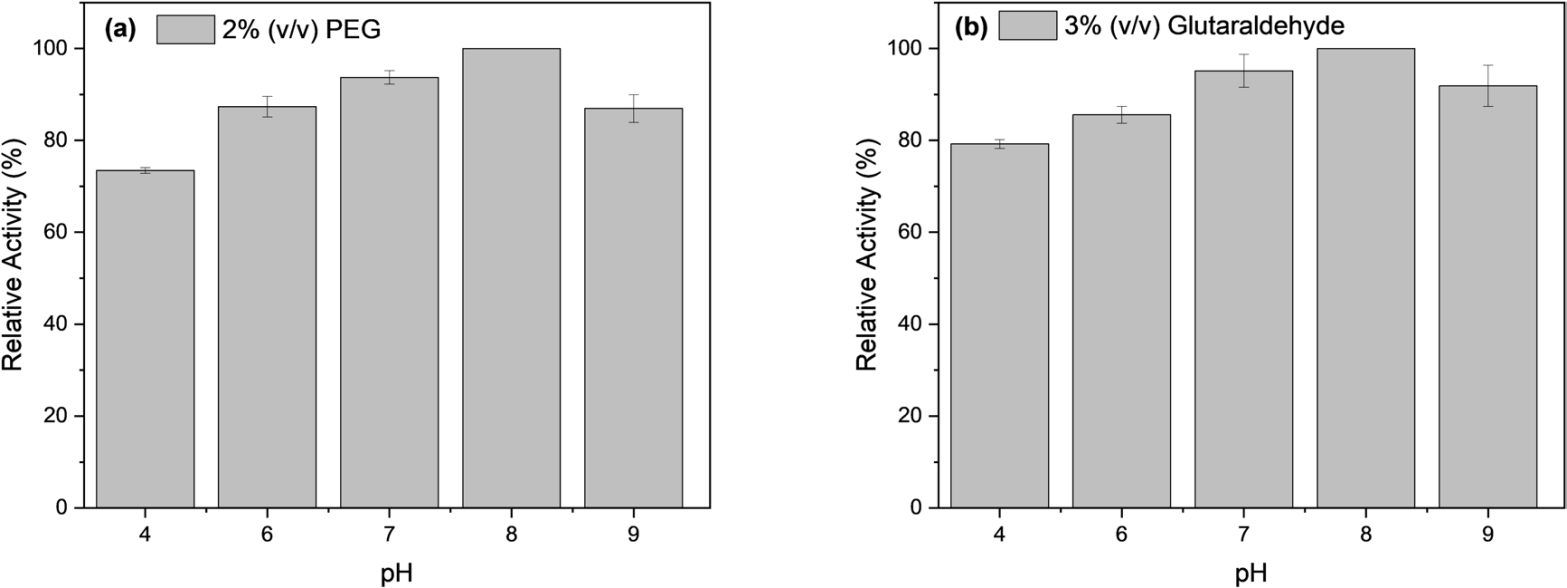
Percentage activity recovery at 2% PEG (**a**), and at 3% GA (**b**) of different pH

The results in Fig. 4a, b convey the increase in lipase activity from pH 4 until pH 8, followed by the trend where lipase performance starts to show a decline upon reaching pH 9. While comparing the PEG and GA-treated lipase hybrid nanoflowers, both of them show the highest lipase activity performance at pH 8, which also indicates the lipase enzyme is deemed to be favorable at this slightly basic condition. Overall, the 3% (v/v) GA-treated lipase hybrid nanoflowers display higher lipase performance than that treated with 2% (v/v) PEG in terms of specific activity over the range of pH 4 – pH 9, where the highest specific activity is computed to be 40,245 U/g and 39,288 U/g respectively at pH 8.

**Fig. 5:**
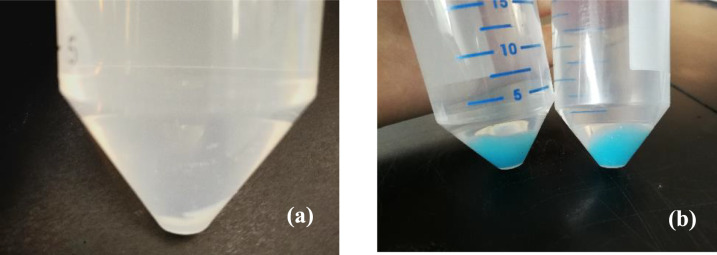
The blue precipitate formed at pH 4 (**a**), and other pH (**b**)

On the other hand, it is observed that the hydrolytic activity of PEG and GA-treated lipase hybrid nanoflowers is relatively low at pH 4. During the experiments to synthesize the treated-lipase hybrid nanoflowers, it is noticed that the copper (II) sulfate is highly soluble at pH 4 sodium phosphate buffer, instead of forming insoluble Cu_3_(PO_4_)_2_ precipitate. Therefore, only little amount of precipitate is formed (Fig. 5a), as compared to the others in Fig. 5b. This phenomenon can be explained by Aksu [40], where the solubility of Cu_3_(PO_4_)_2_ is dependent on the pH, the concentration of copper (II) ions, as well as the concentration of phosphate ions. The precipitation of Cu_3_(PO_4_)_2_ might be difficult to occur if either the concentration of copper (II) ions or phosphate ions does not satisfy the requirements at a certain pH. On the other hand, as copper (II) ions are predominant at lower pH, most of them are likely to dissolve in the aqueous medium rather than forming a precipitate with phosphate ions, which is probably attributed to the reduction in blue colour intensity of the precipitate formed as shown in Fig. 5a. As a result, the immobilization of lipase is affected due to insufficient Cu_3_(PO_4_)_2_ support. This is further evidenced by the work of Altinkaynaka et al. where the result reveals that no nanoflowers formation occurs at pH 4 and pH 5 [41].

### Comparison of treated and untreated lipase hybrid nanoflowers

While most of the immobilization methods are found to cause a significant reduction in enzymatic activity when compared to free lipase despite the superior improvement in stability, it is crucial to examine the ability of the immobilized lipase within hybrid nanoflowers to maintain the lipase hydrolytic activity. From the previous section, it is known that the optimum concentration of lipase, PEG, and GA are 0.2 mg/mL, 2% (v/v), and 3% (v/v), respectively.

**Fig. 6:**
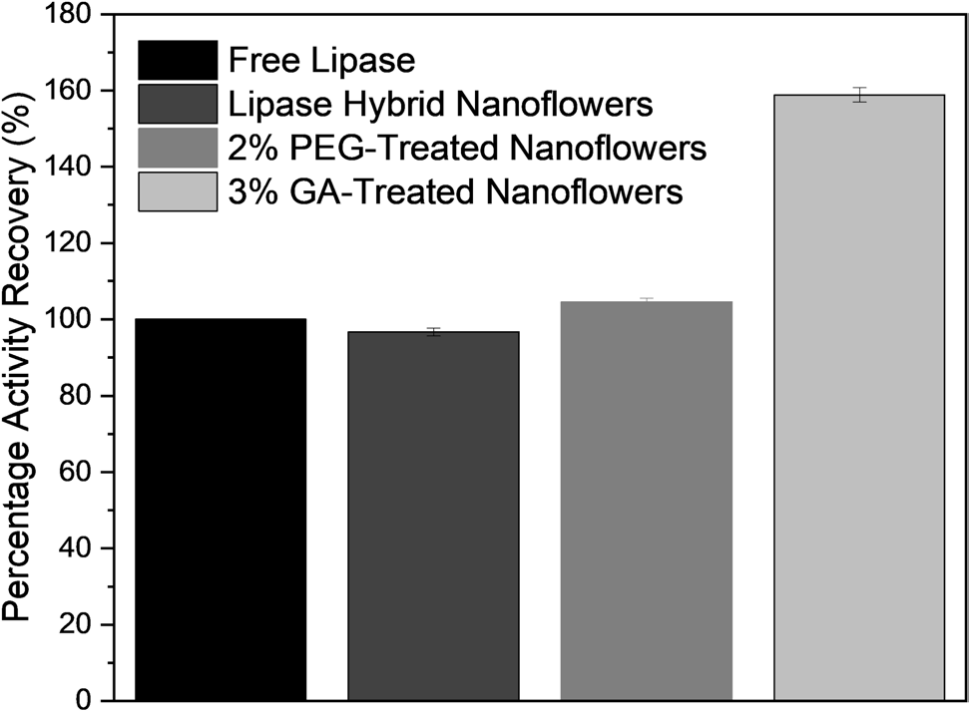
Percentage activity recovery at different conditions

To allow a clearer picture view, a graph showing the comparison among free lipase, lipase-copper hybrid nanoflowers without treatment, and lipase-copper hybrid nanoflowers pretreated with optimum concentration of PEG and glutaraldehyde is plotted in Fig. 6. By taking free lipase as a reference, the hydrolytic activity of conventional lipase hybrid nanoflowers is observed to be slightly lower than that of free lipase, as described in a previous study by Ge et al. [15]. The loss in activity of conventional lipase hybrid nanoflowers is shown to be compensated through the incorporation of PEG and GA. The additional treatment of hybrid nanoflowers with PEG and GA is not only capable of retaining the initial hydrolytic activity demonstrated by free lipase but also exhibits further enhancement in the activity performance, particularly GA, where the GA-treated hybrid nanoflowers manage to impart a maximum 60% increment of the initial free lipase activity, which is deemed to be much higher than PEG.

### Characterisation of hybrid nanoflowers

#### Scanning electron microscopy (SEM)

**Fig. 7:**
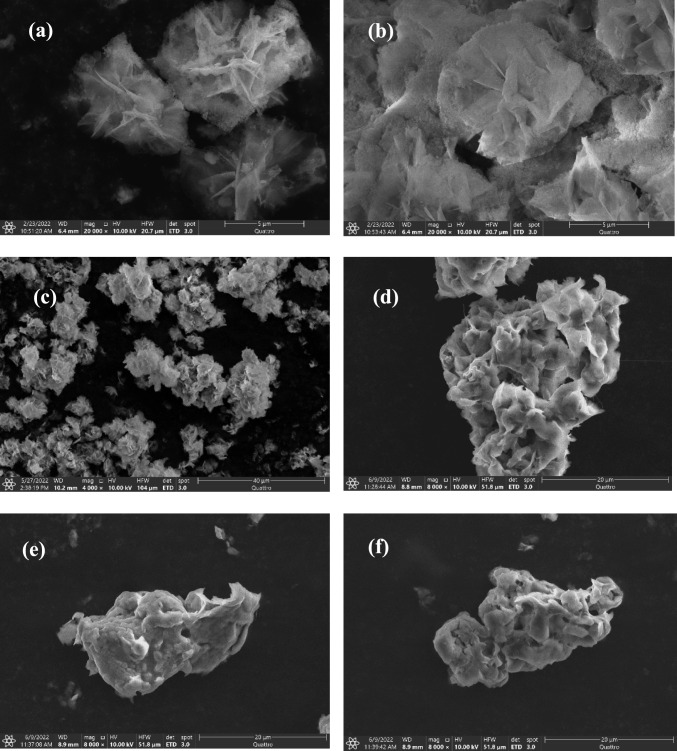
SEM image for lipase hybrid nanoflowers without treatment (**a**, **b**), 2% PEG-treated lipase hybrid nanoflowers (**c**, **d**), and 3% GA-treated lipase hybrid nanoflowers (**e**, **f**)

The morphology of PEG and GA-treated lipase hybrid nanoflowers is characterized via scanning electron microscope and compared to the conventional lipase-copper hybrid nanoflowers without treatment obtained by Anboo et al. as given in Figs. 7a, b [38].

By observing Figs. 7c, d, the lipase hybrid nanoflowers modified with 2% (v/v) PEG seem to be capable of retaining the typical flower-liked structure. However, when the SEM is magnified in Figs. 7c, d, it is obvious that the nanoflowers matrixes are attached forming a large complex. This phenomenon is due to the precipitation effect contributed by PEG, where this non-activated PEG with low molecular weight of 600 Da probably forms intermolecular hydrogen network with the hybrid nanoflowers matrixes.

In Fig. 7e, f, the nanoflowers structure of 3% (v/v) GA-treated lipase hybrid nanoflowers seems like losing the initial blooming flower shape when compared to the conventional hybrid nanoflowers. Instead, it imparts a more complex and rigid structure which is probably caused by the polymerisation of glutaraldehyde forming extensive multi-point cross-linkages with enzyme molecules, that often results in the formation of large molecular weight precipitates [31]. However, the void spaces are still observable in the structure. Similar condition was encountered in previous work by Soni et al. [32] where the morphology of glutaraldehyde-treated nanoflowers is observed to agglomerate in mass.

Overall, the implementation of both PEG and GA is deemed to affect the morphology of hybrid nanoflowers, especially for GA, since theoretically covalent bond is much stronger than a hydrogen bond, which can reduce the flexibility of the structure. Surprisingly, when looking back to Fig. 6, the catalytic activity of GA-treated hybrid nanoflowers is well-performed despite the disrupted flower structure, which might be probably due to the ability to preserve some void spaces, so that the decrease in surface area of contact is not too high. Therefore, it can be presumed that the effect of morphology becomes insignificant when the advantages of GA dominate.

#### Fourier transform infrared (FTIR) spectroscopy

**Fig. 8:**
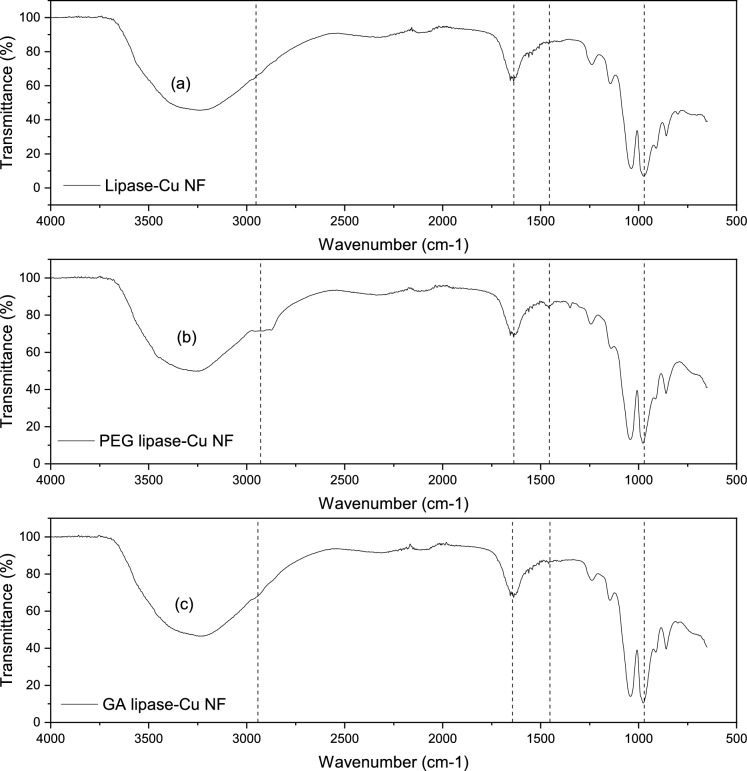
FTIR plot of lipase hybrid nanoflowers without treatment (**a**), PEG-treated lipase hybrid nanoflowers (**b**), and GA-treated lipase hybrid nanoflowers (**c**)

The lipase-copper hybrid nanoflowers with and without treatment are monitored by FTIR to examine the vibration and stretching of certain functional groups, as depicted in Fig. 8. The spectrum region from 1700 cm^−1^ to 1300 cm^−1^ is due to the presence of a protein secondary structure. The peak between 1700 cm^−1^ and 1600 cm^−1^ indicates the stretching of amide I, which is mainly caused by the C$=$O bond of amide, whereas wavelength from 1600 cm^−1^ to 1300 cm^−1^ shows the vibrations attributed to amide II (bending of N$-$H), amide III, and possibly some amino acid side chains [42–44]. All three samples impart the specified characteristics, which confirms the encapsulation of lipase enzymes within the nanoflowers. A strong stretching peak is noticed at a wavelength of 1025 cm^−1^ – 870 cm^−1^, for spectra (a), (b), and (c), which notify the presence of phosphate group (P$-$O). According to Yu et al. [11], Cui et al. [45], and Zhang et al. [35], there is another spectrum region caused by the bending vibrations of bridging O$-$P$-$O at 630 cm^−1^–550 cm^−1^. This characteristic is not found here due to the limitation of the spectrum range to 650 cm^−1^.

### Bioplastic degradation

**Fig. 9:**
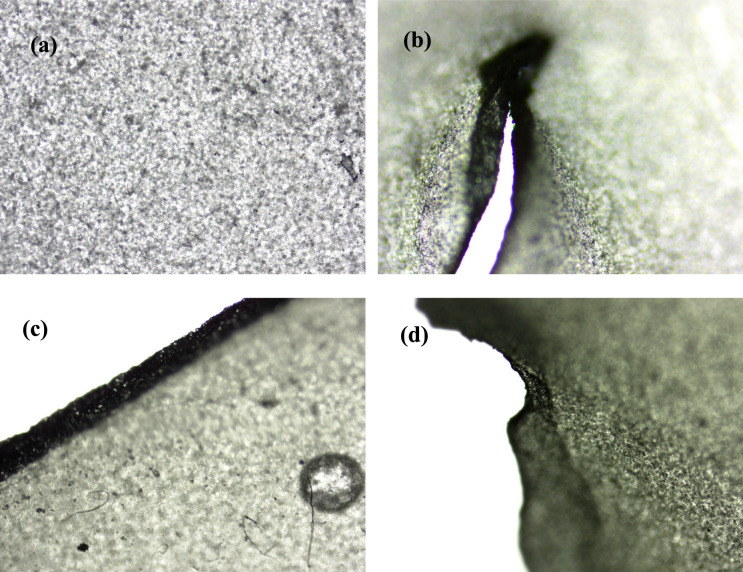
Microscopic images of bioplastic at 40 $\times$ magnification (**a**, **c**) Before degradation (**b**, **d**) After degradation

**Fig. 10:**
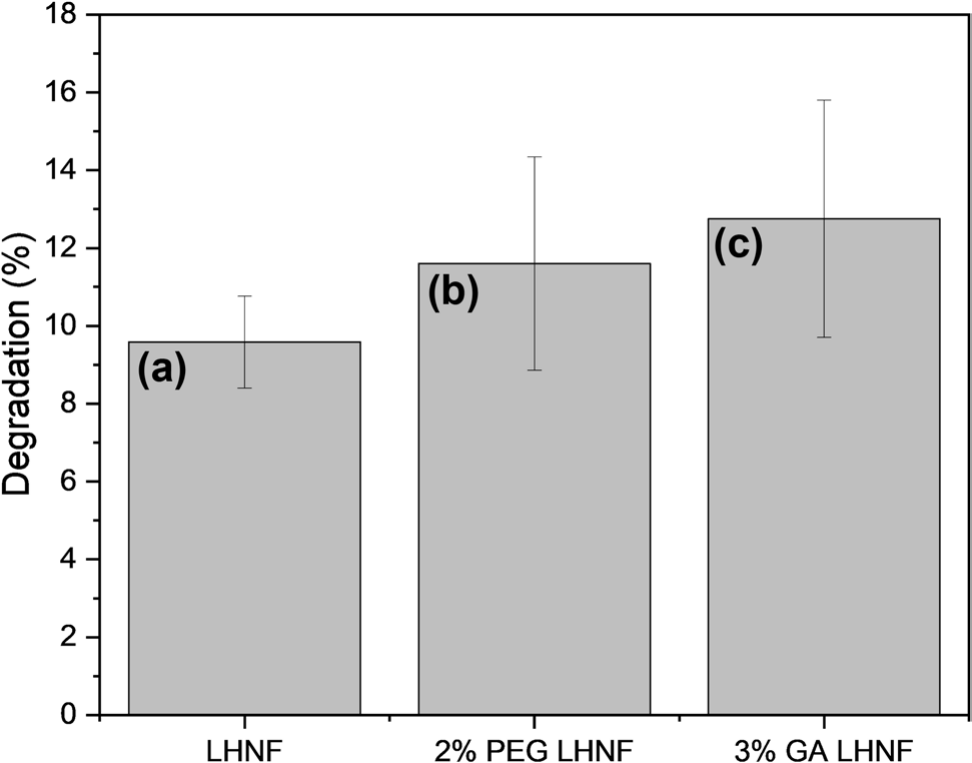
Degradation enhancement by **a** Untreated lipase hybrid nanoflowers (LHNF) **b** 2% PEG treated LHNF **c** 3% GA treated LHNF

**Fig. 11:**
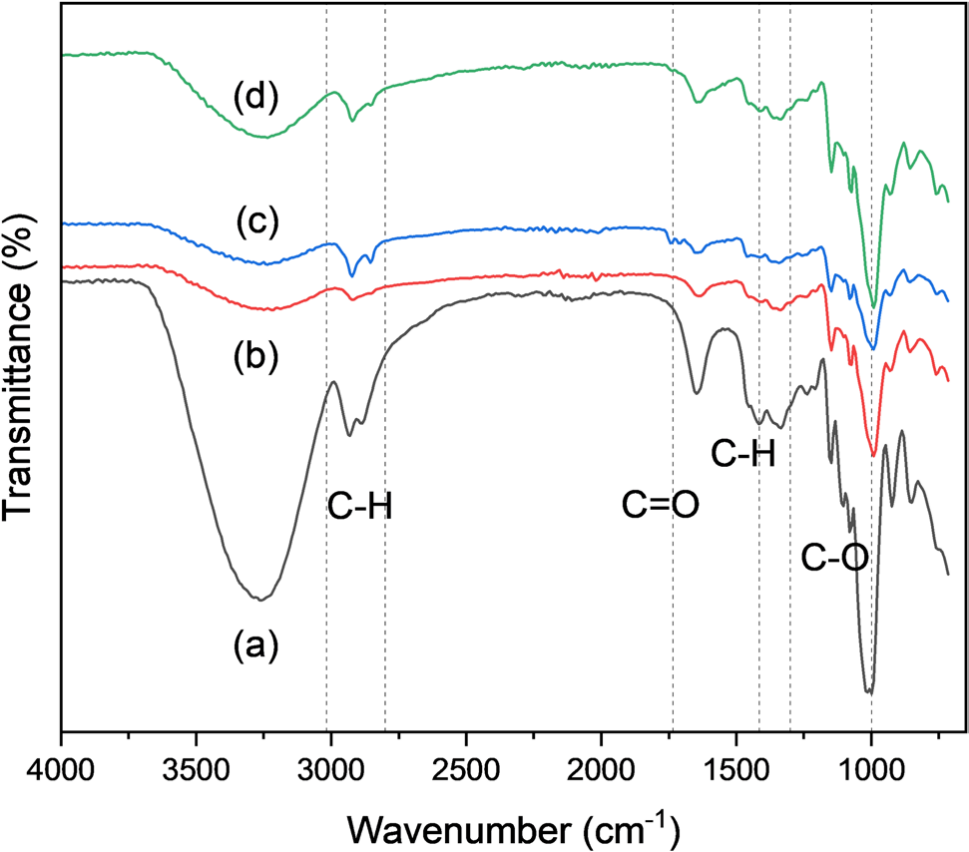
FTIR spectra of bioplastic **a** Before degradation **b** After degradation by untreated LHNF **c** After degradation by 2% PEG treated LHNF **d** After degradation by 3% GA treated LHNF

Bioplastic degradation tests are carried out for untreated, 2% PEG treated, and 3% GA treated lipase hybrid nanoflowers. Figure 9 shows the morphology of the bioplastic before and after degradation. Initially, the bioplastic has a complete structure with relatively neat edge. Cracks and irregular edges are observed to appear after degradation. Using blank as control, the application of untreated, 2% PEG treated, and 3% GA treated lipase hybrid nanoflowers has further improved the bioplastic degradation by 9.6%, 11.6%, and 12.8% respectively at day 7 (Fig. 10). This further highlights the greater performance of GA than PEG as enhancer. The FTIR spectra of corn starch based bioplastic before and after degradation are illustrated in Fig. 11. The peaks at range 3000–2800 cm^−1^ and 1414 cm^−1^ are due to C$-$H (starch), 1750–1700 cm^−1^ are attributed to C$=$O (esters and carboxylic acids), and 1300–1000 cm^−1^ are contributed by mixtures of different bondings, which include C$-$O of esters and carboxylic acids. The degradation of bioplastic by lipase nanoflowers are primarily through the hydrolysis of ester bonds, resulting in more carboxylic acids formation, which is obvious especially for 2% PEG treated and 3% GA treated lipase hybrid nanoflowers at 1700–1730 cm^−1^. The decrease in intensity of C$-$H bands also indicates the break down of bioplastic into smaller chains.

## Conclusion

The study successfully investigates the effect of polyethylene glycol (PEG) and glutaraldehyde (GA) on the performance of lipase hybrid nanoflowers. Through the Bradford assay, the optimum lipase concentration was determined to be 0.2 mg/mL in the presence of 120 mM copper (II) sulfate, which corresponds to the saturation point of the hybrid nanoflowers. The encapsulation yield of lipase within the nanoflowers increased up to this optimal concentration before starting to decline. The effects of PEG and GA concentrations, as well as pH, on the enzymatic activity of the lipase hybrid nanoflowers were thoroughly evaluated. The optimal concentrations of PEG and GA were found to be 2% (v/v) and 3% (v/v), respectively. It was observed that lower concentrations were ineffective in enhancing the lipase activity, while higher concentrations resulted in inhibition. Both PEG and GA-treated lipase hybrid nanoflowers demonstrated the highest enzymatic activity at pH 8.

The comparative study between free lipase, untreated hybrid nanoflowers, and treated hybrid nanoflowers revealed that glutaraldehyde was a more effective enhancer of lipase activity than PEG. Specifically, the GA-treated hybrid nanoflowers exhibited a 160% increase in lipase activity compared to the free enzyme, highlighting the superior performance of GA as an enhancer. Furthermore, the morphological analysis of the hybrid nanoflowers indicated that while GA treatment caused significant changes in the flower-like structure, it did not hinder lipase activity, possibly due to the preservation of void spaces within the structure. In contrast, PEG-treated nanoflowers maintained their flower-like morphology but exhibited less pronounced improvements in activity. This was further supported by bioplastic degradation experiments where GA showed higher enhancement in degradation (12.8%) than PEG (11.6%).

In conclusion, this study demonstrates that the incorporation of PEG and GA can significantly enhance the enzymatic performance of lipase hybrid nanoflowers, with glutaraldehyde offering superior enhancement compared to PEG. The results underscore the potential of GA as a promising enhancer for the production of highly active lipase hybrid nanoflowers, which may have important implications for biocatalysis applications.

## Data Availability

No datasets were generated or analysed during the current study.
